# Investigating the application of one piece flow from lean manufacturing in the construction delivery of mass housing projects

**DOI:** 10.1038/s41598-025-19779-w

**Published:** 2025-09-30

**Authors:** Dina Atef Saad, Moheb Habib, Azza Abou-Zeid

**Affiliations:** 1 Structural Engineering Department, Faculty of Engineering, Cairo University, Giza, Egypt; 2 Faculty of Engineering, Cairo University, Giza, Egypt

**Keywords:** Real estate housing, Lean construction, Economic evaluation, Batch construction, Single-piece-flow, Engineering, Civil engineering

## Abstract

Mass-housing projects (MHPs) are often delivered using mass construction to save time and cost. However, it actually leads to large work in progress (WIP), delays, and cost overrun. Moreover, to finance mass construction works, housing developers often sell off-plan large number of housing units leading to financial losses especially during economic downturns. Accordingly, this research evaluates the effectiveness of adopting the lean concept “One-Piece Flow (OPF)” for delivering MHPs instead of mass construction. OPF-based construction relies on batch production which helps reduce WIP, production cycle time, and rework. A comparative analysis, using an actual case study, was conducted between mass construction and OPF-based construction in terms of performance and economic worthiness. Moreover, despite completing the project in more time and cost, the results showed that the OPF-based construction delivery achieved, on average, 43% higher profitability and reduced time waste by an average of 47% per building. Thus, this research confirms the potential of adopting OPF-based construction for MHPs.

## Introduction

Mass-housing Projects (MHPs) are development projects that involve mass construction of standardized housing units (i.e., same architectural design) in the same location, similar to mass production in manufacturing. The housing units could be multi-story buildings or towers, semi-detached and/or detached residences, or a combination of these^1,2^. Accordingly, they are different from one-off building projects due to the repetition of work across multiple identical units^3^.

Timely construction delivery of MHPs is usually an essential requirement, whether they are public or private development projects. Accordingly, several developers opted for mass construction by building large batches of construction work (phases, buildings) at the same time in MHPs due to their repetitive nature, and to presumably save time and benefit from the large economies of scale^2^. This has been witnessed in developing countries where governments are required to rapidly deliver mass-housing development projects for low- and medium-income householders to accommodate fast population growth; examples include Iran, Turkey, Egypt, Ghana, Latin American countries^2,4–8^. Mass construction is also commonly used in delivering private real estate residential developments^6^, where real estate developers usually aim to maximize their profit by increasing sales revenues and minimizing construction costs. Despite its widespread use, the construction of multiple units at the same time using mass construction can lead to several problems, including poor site management, material procurement problems due to overlapping activities (e.g., pouring concrete), over-allocation of resources to non-value adding activities. Furthermore, quality may be compromised, and significant reworks may be required due to the late detection of defects across a large number of constructed units^9,10^. Consequently, this approach can cause delays, cost overruns, and financial strain due to the need to cover increased construction costs and finance the large volume of concurrently executed work. In addition, it can result in profit loss from selling a large number of units off-plan, which means selling designed units before construction begins or during construction to secure funds for the mass construction works. In times of economic instability—marked by fluctuations, surging energy prices, and high inflation—consumers often turn to real estate as a means of preserving the value of their money. This behavior increases demand for housing units, subsequently leading to a rise in their prices^11^. However, real estate developers may risk of losing profits due to selling a large number of housing units at low prices that didn’t account for inflated construction costs over time. Consequently, the financial feasibility of MHPs’ depends heavily on the delivery method, the associated sales strategy, and the broader economic conditions that influence consumer investment behavior in the housing market.

Accordingly, this research is investigating, using a real case study of a MHP, the impact of mass construction on: (1) project duration and cost, (2) work-in-progress (WIP), which captures unit idleness due to delays, and (3) project profitability. Moreover, due to the resemblance between mass construction of repetitive units and mass production in manufacturing — known as “batch-and-queue”^1,5,9^ — this research proposes a construction delivery method that adopts the lean manufacturing concept “One-piece Flow” to overcome the drawbacks of mass construction in MHPs. The following section describes the One-piece Flow concept and its application in the construction industry.

## Literature review

Several research efforts studied the factors affecting the construction performance in terms of time, cost, and quality in the one-off construction projects^12–14^. However, few research efforts discussed the impact of the construction delivery method on the construction performance in MHPs. For example, Faraji et al.^4^ studied the factors influencing the costs of mass housing projects and their subsequent impact on affordability in developing countries. Noorzai and Golabchi^7^ and Noorzai et al.^15^ discussed the potential of modular construction and prefabricated elements as alternatives to conventional on-site methods, enabling faster project delivery and minimizing rework in MHPs. Lee et al.^16^ studied the benefit of utilizing modular construction in mass rental housing projects for reducing time and cost. Balouchi et al.^17^ identified the significant causes of rework in MHPs in Iran, including the lack of proper plan or method statement, which leads to improper commissioning or execution of a part of an activity. Heravi and Jafari^8^ studied the factors contributing to quality issues and the associated costs in MHPs in Iran, including failure to analyze non-conformances and implement corrective actions, failure to inspect and ensure precision and accuracy during execution, and failure to address the non-conformances closer to project delivery, which leads to higher expenses. Ahadzie et al.^2^ discussed the critical factors that contribute to the successful completion of mass-housing projects, including environmental impact, quality, customer satisfaction, cost of individual units, and project duration. Despite the usefulness of the aforementioned efforts, none has examined the impact of the construction delivery method in MHPs on the project’s economic viability and performance in terms of time, cost, and unit idleness (i.e., units remaining idle due to delays or interruptions).

### Lean construction: one-piece flow

Lean manufacturing, led by Toyota Production, has been advocated to overcome the drawbacks or wastes of mass manufacturing, including: large WIP, over production, late detection of errors, and large inventories leading to increased costs and longer durations^18^. Despite the physical differences between manufacturing and construction in terms of the features of the end output — manufacturing is a factory-based production with a product as the end output, while construction is a site-based production with a one-of-a-kind project as the end output^19–21^ — there is resemblance between manufacturing and construction of repetitive units^9^. Manufacturing is a machine-driven process in which products move from one machine to another, while construction is a resource-driven process in which resources flow from one repetitive unit to another^22,23^. Accordingly, several lean manufacturing concepts have been adopted in the construction industry^9^. Further discussion on the differences between lean manufacturing and lean construction, and on the adaptation of lean philosophy to construction, can be found in the technical report by Koskela^21^ and in the Construction Industry Institute report^9^.

In lean construction, minimizing waste and non-value-adding work are measures of construction performance^24,25^. Therefore, among the lean manufacturing concepts that have been adopted in the construction industry is the “One-piece flow, OPF” concept, also known as “single-piece flow” or “continuous flow”^9^. One-piece flow is the production of one unit or small-size batches at a time to maintain the smooth flow of units without any interruption, thereby minimizing the wastes and the capital needed for mass production^9,26^. Similarly in the construction industry, delivering projects in small-size batches helps reduce inventories on site and WIP, in terms of idle units either waiting for design, availability of resources, or funds to be completed^22^. Reducing WIP is essential to minimize non-value-adding work and the vulnerability of partially constructed works to damage while waiting idle for completion^26,27^. In addition, construction in small batches helps with the early detection of defects and prevents their reoccurrence in the succeeding batches^28,29^.

Few research efforts studied delivery of construction projects using batch-production or one-piece flow, especially repetitive ones due to their resemblance to manufacturing of identical units. For example, Ward and McElwee^29^ investigated the impact of batch size on the duration of completing the finishing works of repetitive hotel rooms. Shim^28^ discussed the benefits of using small and matched batch sizes to reduce project duration in repetitive construction projects using Monte Carlo simulation. Shim and Kim^30^ developed a batch-based repetitive scheduling method and examined the impact of unbalanced batch sizes among activities. Saad at al.^5^ developed a multi-objective optimization model for scheduling repetitive projects using pull and batch production to determine the optimum number of batches and crews (travelling from one repetitive unit to another) that minimize cost, time, and WIP in terms of unit cycle time. Khan and Leicht^19^ conducted semi-structured interviews to explore the benefits and challenges of implementing flow planning in construction. Key benefits included improved efficiency, better site management, and early project completion, while recurring challenges involved design issues, limited experience with flow concepts, and reliance on traditional scheduling methods.

Despite the usefulness of the aforementioned efforts, they all discussed the impact of batch production on reducing construction cost and time. However, none of the existing research efforts has investigated the difference between mass construction and OPF-based or small-size batch construction in terms of construction performance and economic worthiness, particularly from the perspective of the MHP developers.

## Research objective and scope

**Fig. 1:**
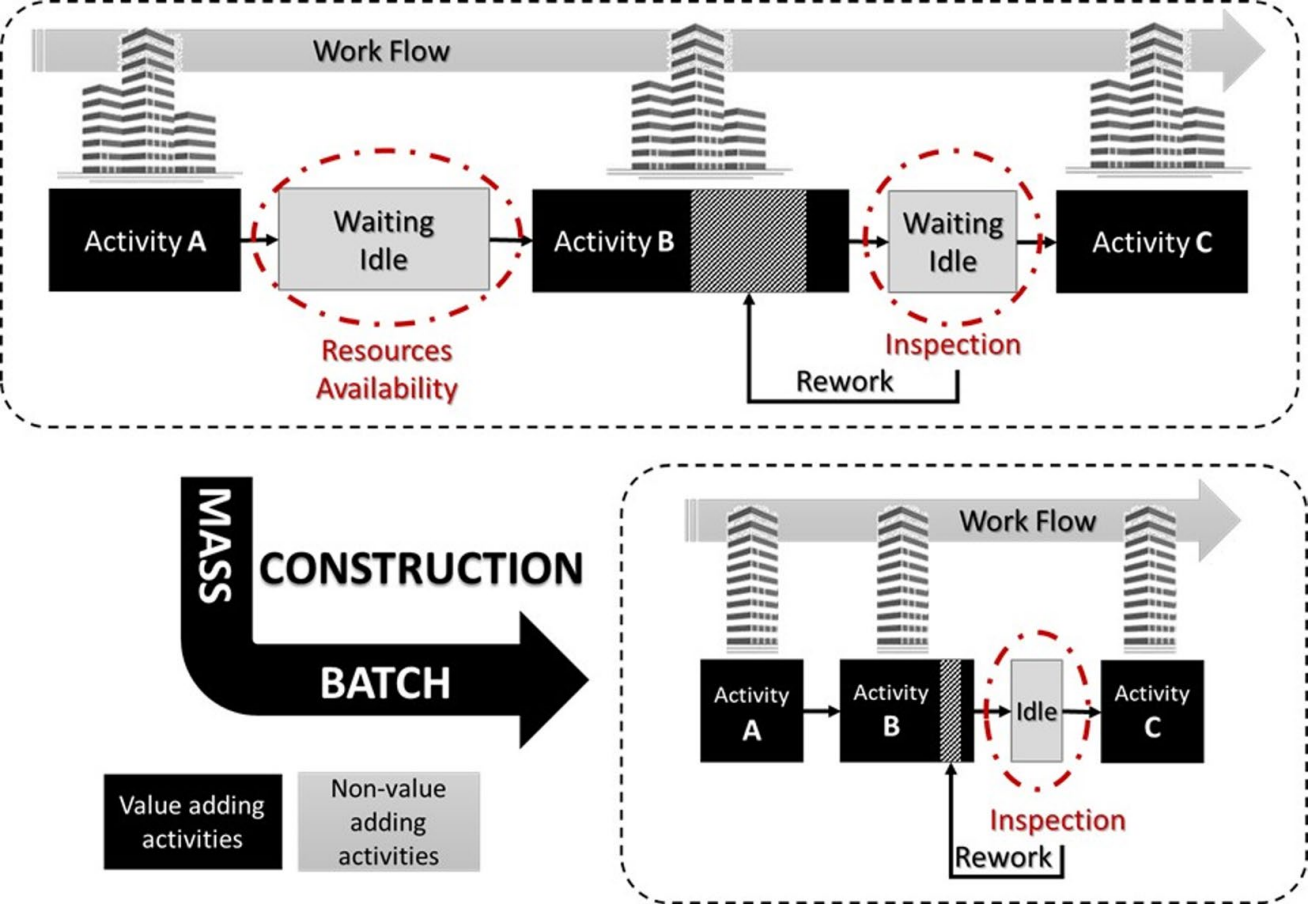
Work flow map in case of mass construction and OPF-based construction.

This research proposes an OPF-based construction delivery method that adopts the lean concept “One-piece Flow (OPF)” in delivering MHPs using small-size batches of housing units. The proposed delivery method allows the smooth flow of resources between batches, minimizing work-in-progress as well as costly and time-consuming rework. Figure 1 shows an illustrative map of the expected work flow differences between the proposed OPF-based construction delivery and mass construction of multiple buildings, in terms of time waste, and non-value-adding activities. In mass construction, as opposed to OPF-based construction, units remain idle for longer periods, either waiting for the availability of resources or for inspection. The figure also shows the expected significant time wasted in rework of mass units in cases where defects are detected during inspection. Thus, the OPF-based construction can help achieve better quality due to early detection of defects before proceeding to the subsequence batches, increase productivity by reducing rework, reduce material inventory and over-procurement, and WIP in terms of the unit cycle time. Moreover, the proposed OPF-based construction allows selling housing units in batches, which can help absorb fluctuations in prices or economic downturns. To demonstrate these potential benefits, this research conducts a comparative analysis between the proposed method and the conventional mass construction method, as described in the following section.

This research focuses only on conventional on-site construction methods. Although modular construction, or industrialized building (IB), has been advocated as a lean approach to minimize construction waste and optimize time and cost^7^, it requires standardized design, standardized production methods of prefabricated structural system, and large-scale development^16^, which are not commonly used in MHPs, especially in developing countries^31^.

## Research methodology

**Fig. 2:**
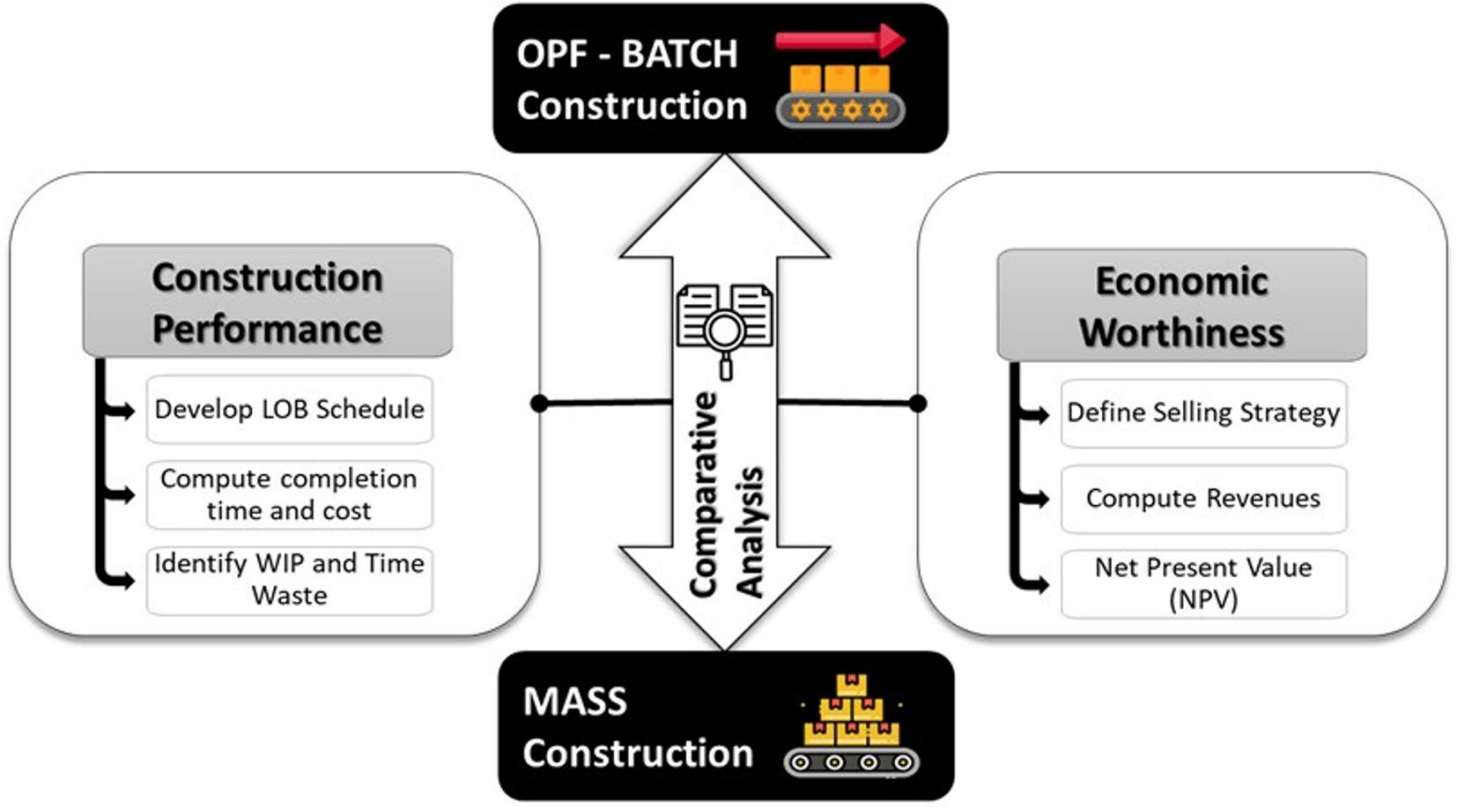
Research framework.

This research relies on the concept of case-based research, where a single real case study is used to explore or investigate a research idea when there is no prior empirical evidence supporting it. Case-based research has also been previously used to prove the applicability of lean construction theories for practitioners to implement^32,33^. Following this notion, a real residential mass-housing project in Egypt was used as a case study in this case-based research. Using this study, a comparative analysis was conducted to quantitatively assess the efficacy of the proposed OPF-based construction in comparison to mass construction, as shown in Fig. 2, considering both construction performance and economic worthiness. For the construction performance, the comparison considered the total completion time and cost, as well as time waste in each building unit in terms of rework and delays. Moreover, since MHPs consist of repetitive units (e.g., floors, buildings, houses), repetitive line-of-balance (LOB) schedules were developed^34^ to visually track delays through the slope of the flow lines (i.e., progress rates) and to identify the specific points in time when the delays occurred. Repetitive scheduling is well aligned with the lean concept of One-piece Flow, as it allows resources to flow continuously from one repetitive unit to another, unlike the critical path method^25^. Accordingly it is recommended for scheduling the construction activities of MHPs.

For economic feasibility, the comparative analysis includes an economic evaluation of the project’s cash flows over a defined time horizon. The cash flows include the construction costs, additional costs due to rework, and the sales revenues, which vary according to the adopted delivery method and associated selling strategy, as previously described. The following section describes the case study used in the comparative analysis.

### Actual case study description

The construction project under study is for a residential complex consisting of six identical buildings in Egypt. The project started in 2015 and was completed in 974 days. It was delivered using mass construction, with the six buildings constructed simultaneously to presumably save time and costs. The actual data and durations were collected from project documents (as-built schedules and site reports), and through interviews with site engineers on-site. A detailed description of the study has been reported in Saad et al.^35^. During construction, Egypt witnessed several economic fluctuations, including the first currency devaluation in November 2016 and removal of fuel subsidies, which led to sudden surge in wages and material prices and wages beyond risk contingencies, resulting in significant cost overruns.

In housing developments, as previously mentioned, developers commonly sell units off-plan to consumers (house buyers) ahead of construction to cover expenses. In the project under study, the developer sold a large percentage of the units at the old prices, based on the estimated construction costs before the devaluation and removal of subsidies. Due to the sudden increase in prices, the developer unexpectedly faced a funding shortage to cover the increased construction costs. As a result, the work on site was interrupted, leading to large WIP (i.e., idle, partially constructed units with no progress) and time delays. This problem was observed across the Egyptian housing market during this period of economic instability.

Moreover, the developer lost a significant amount of profit due to missing the opportunity to sell the units at the new prices. Due to the economic instability, consumers preferred to invest in the real-estate housing market to protect their savings against the drop in the currency value. Consequently, housing unit prices increased enormously due to the increase in demand compared to the limited available supply. Most of the problems encountered in this project could had been avoided if the project had been delivered in small batches rather than one large batch of six buildings. Although this case study dates back to the period between 2015 and 2017, the current state of the Egyptian Economy resembles that period. Accordingly, the study remains relevant to the present time. Moreover, mass construction using conventional methods is still used in delivering mass housing projects in Egypt and similar developing countries.

### Comparative analysis: OPF-based construction vs. mass construction

To evaluate the feasibility of the proposed OPF-based construction delivery against the mass construction delivery used in the actual case, two what-if scenarios were developed. In both scenarios, the six buildings are delivered in three successive batches of two buildings at a time instead of delivering all six units simultaneously. In the first what-if scenario, it is assumed that zero waste (delays and rework) will occur, based on the assumption that batch construction allows early detection of errors and thus prevents their repetition in later batches. Moreover, it helps alleviate financial problems since there is no huge volume of work being executed simultaneously. In the second what-if scenario, it is assumed that waste will occur in the first batch, then gradually decrease in the later batches due to the learning effect and early detection of errors. The first scenario can be considered as an ideal or optimistic case, while the second scenario represents a more conservative one. A comparison among the three cases (actual case, Zero-waste OPF-based delivery, and OPF-based delivery) was conducted with respect to: (1) construction performance, and (2) economic feasibility.

#### Construction performance comparison: project duration and time waste

In this comparison, the performance is in terms of project completion time and time waste due to rework or interruptions. As previously mentioned, a repetitive schedule was developed for each building in all three cases. To facilitate developing the repetitive schedules, activities of all main elements (e.g., slabs) in each floor were grouped — considering the logical sequence between them — into one collective repetitive activity, “Floor Completion”. The duration of this activity was calculated as the sum of durations of all sub-activities on the critical path (e.g., formwork shuttering, reinforcement, pouring concrete). However, in lean philosophy, it is preferable to rely on durations estimated from actual progress on site rather than planned durations^36^. Therefore, the duration of executing each floor was computed from the difference between the actual pouring dates of every two successive slabs, as shown in the sample presented in Table 1 below.

**Table 1:** Least duration for completing each floor in each building in the case study.

Floor	Floor slab	Buildings						Least duration ($\:{{L}{D}}_{{f}}$)
		Actual duration ($\:{{A}{D}}_{{f}}$)						
		1	2	3	4	5	6	
0	Foundation	57	103	72	96	38	45	38
1	Basement slab	99	74	75	71	91	97	71
2	Ground slab	43	55	25	57	58	57	25
3	First slab	48	48	30	40	43	66	30
4	Second slab	41	44	31	45	48	65	31
5	Third slab	43	35	27	42	31	59	27
6	Fourth slab	52	68	28	60	43	67	28
7	Fifth slab	65	54	27	61	57	58	27
8	Sixth slab	42	33	27	37	63	41	27
9	Seventh slab	32	56	36	49	48	66	32
10	Eighth slab	40	41	48	62	41	54	40
11	Ninth slab	66	38	22	43	62	85	22
12	Tenth slab	45	31	39	34	40		31
13	Eleventh slab	32	55	28	91	37		28
14	Roof slab	19	62	45	119	68	39	19
Delays due to defected work in each building		93	82	42	163	137	87	
Delays due to financial problems in each building		155	239	42	268	155	295	
Total delay/building		248	321	84	431	292	382	

Delays are often computed as the difference between the actual duration and the planned duration. However, to be aligned with the lean philosophy, the actual time delay (waste in time) was measured as the difference between the actual duration and the least possible duration that could be achieved under site conditions for a given floor (i.e., the best performance achievable on site, eliminating any errors). Table 1 below shows the least possible duration ($\:{\varvec{L}\varvec{D}}_{\varvec{f}}$) for each floor. For example, the least duration across the six buildings for completing 2nd floor was 25 days, while for the 4th floor it was 31 days. The table also shows the total delay encountered per building, which is the sum of delays in each floor, as formulated in Eq. (1) below. Based on the actual data collected on site, as described in the next subsection, the total delay was classified to delays due to defects and those due to financial problems.

1 $$\:{DLY}_{B}=\sum\:_{f=1}^{N}({AD}_{f}-{LD}_{f})$$

Where, $\:{DLY}_{B}$ is the total actual delay encountered per building, $\:{AD}_{f}$_is_ the actual duration for completing a given floor (*f*), and $\:{LD}_{f}$ the least duration that could be achieved per floor under site conditions.

To develop the repetitive schedule for each building, the unit progress rate (*upr*) of the flow line, which represents the flow of crews from one repeated floor to another, was computed as follows^5,37^:

2 $$\:{upr}_{f}\:=\:\frac{1}{{AD}_{f}\:\:}$$

Where, $\:{upr}_{f}$ is the unit progress rate which represents the time consumed to finish one repetitive floor (*f*), $\:{AD}_{f}$_is_ the actual duration for completing a given floor (*f*), and the value (1) represents one unit (i.e., one floor).

#### Mass Construction (Actual Case)

**Fig. 3:**
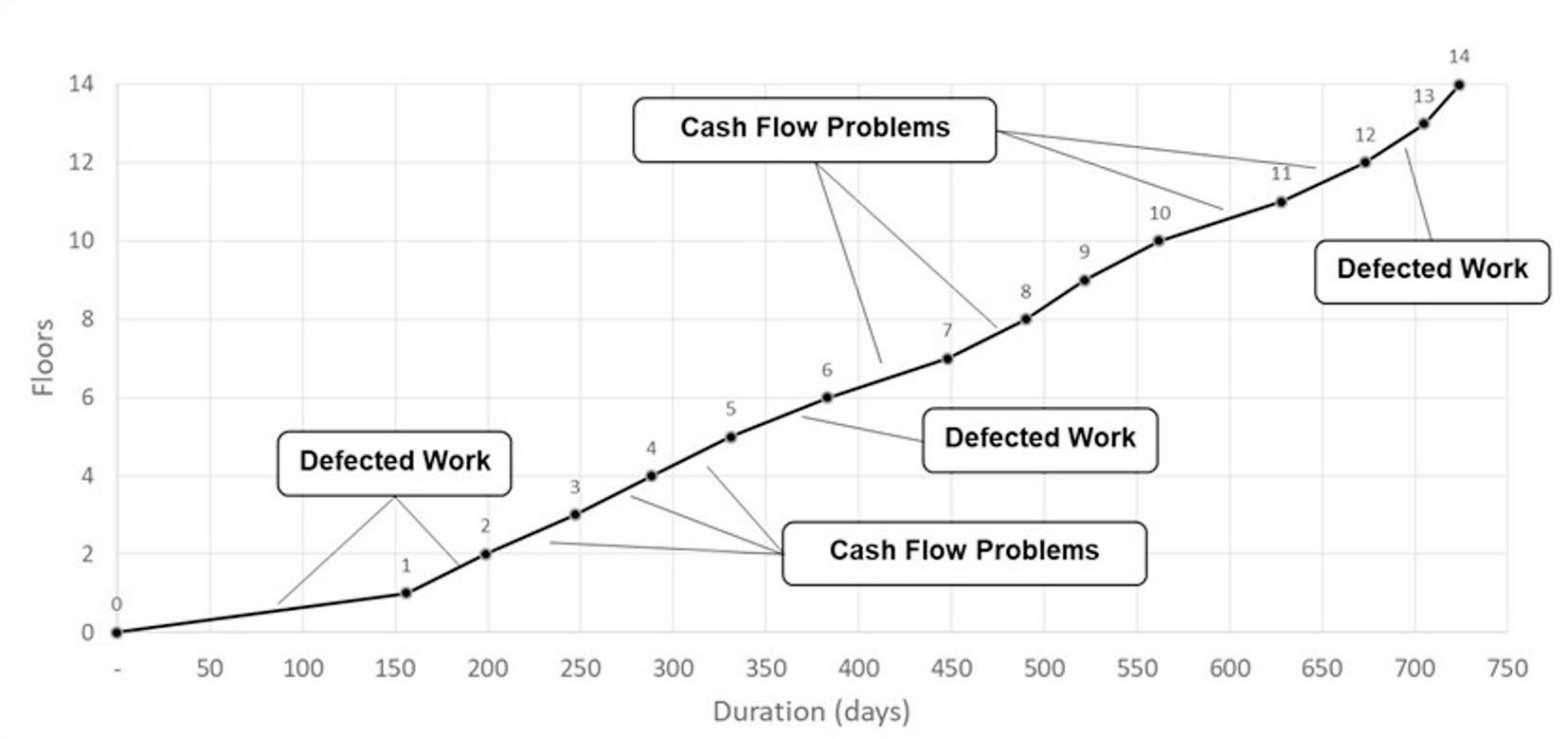
Repetitive schedule for Building 1 in the actual case of mass construction showing the causes of delays.

In the actual case, the total duration of the project was 974 days; however, many delays occurred due to rework resulting from defective work or idleness caused by cash flow problems. Using Eq. (2), a repetitive schedule was developed, as shown in Fig. 3, which illustrates the schedule of building 1 as a sample. It can be visually noted from the flow line slope, the floors where the progress rate was slowed down due to delays and interruptions. For instance, in the first and second floors, a time delay of 65 days was encountered due to rework caused by detected defects in the poured concrete and slab reinforcement. In the 6th floor and 13th floors, time delays of 24 and 4 days, respectively, also occurred due to defects in the slab reinforcement. In addition, idleness was encountered during the construction of the 3rd, 4th, 5th, 7th, 8th, 11th and 12th floors, due to insufficient funds to procure the required material for the six buildings under concurrent construction. The total time delays in each building due to defected work and financial problems are summarized in Table 1.

#### Zero waste OPF-based construction delivery scenario

In this scenario, the buildings are delivered in three successive batches of two buildings at a time. The same subcontractors are employed across all three batches. Accordingly, it can be ideally assumed that zero waste caused by rework of defected work will occur, due to improved learning curve and early detection of errors. Moreover, since only two buildings are constructed at a time, no large volume of work will be running concurrently, and therefore no financial problems or idleness will be encountered. Based on those assumptions, each floor in each building is assumed to be constructed in the least duration ($\:{LD}_{f}$), and thus each building is completed in the least possible duration of 476 days, which is almost 8 months shorter than the actual case. However, using this scenario, the project duration is extended to 1,234 days, due to delivering the project in successive batches.

#### OPF-based construction delivery scenario

In the previous scenario, it might be overly optimistic to assume zero waste and minimum durations for completing each floor in each building. Although such durations were achieved in reality for some floors, as shown in Table 1, assuming them for all floors represents an ideal case. Accordingly, in this scenario, the project is also delivered in three successive batches of two buildings, but under more conservative assumptions. Delays due to defected work and rework are assumed to occur in the first batch, similar to the actual case. However, these defects can be avoided in later floors due to the repetitive nature of the work and the improved learning curve^36^. This assumption is supported by the fact that using the same subcontractor allows problems identified in earlier batches to be alleviated in subsequent ones. Therefore, it is assumed that rework will be reduced in the second batch, and completely eliminated in the third batch. As for financial problems, since the project is not executed through mass construction, delays caused by funding shortages are eliminated.

Based on those assumptions and to facilitate incorporating delays into the scenarios, an average percentage value of the delays that actually occurred in the first batch in comparison with the ideal duration has been computed. This average delay value ($\:\text{\%}{DLY}_{avg}$) was computed using the delay values due to only defected works in each building, previously presented in Table 1, as formulated below in Eq. (3). Accordingly, the duration of any given floor in any given batch is computed by multiplying the least duration $\:{(LD}_{f})$ that could be possibly achieved in each floor by an amplifying factor using the computed average delay value ($\:\text{\%}{DLY}_{avg}$), as formulated below in Eq. (4).

3 $$\:\text{\%}{DLY}_{avg}=\:\frac{\sum\:_{B=1}^{N}\left(\right[{({DLY}_{{B}_{DW}}+IDur}_{B})/{IDur}_{B}]-1)\times\:100)}{N}$$

4 $$\:{Dur}_{{f}_{BT\text{n}}}= {LD}_{f}(1+\text{\%}{DLY}_{avg})$$

Where, $\:{Dur}_{{f}_{BT\text{n}}}$ is the duration of a given floor (*f*) in a building in the a given batch (*BT*_*n*_), $\:{DLY}_{{B}_{DW}}$is total time delay (DLY) due to defected work (*DW*) in a given building (*B*) computed from the sum of delays in each floor as shown in Table 1, $\:{IDur}_{B}$ is the ideal duration of completing a given building (*B*) assuming zero waste, and $\:N$ is the total number of buildings.

Applying Eqs. 3 and 4 to the case study, the resulting value of $\:\text{\%}{DLY}_{avg}$was 22%. This percentage is conservatively assumed to decrease by 50% in the second batch and to reach 0% in the third batch (i.e., 100% of the actual rework delays will occur in the first batch, 50% in the second batch, and 0% in the third final batch). Therefore, the first batch was completed in 579 days, the 2nd batch was completed in 527 days, and the 3rd batch was completed in the least possible duration of 476 days. The overall project duration in this scenario is extended to 1,450 days.

**Fig. 4:**
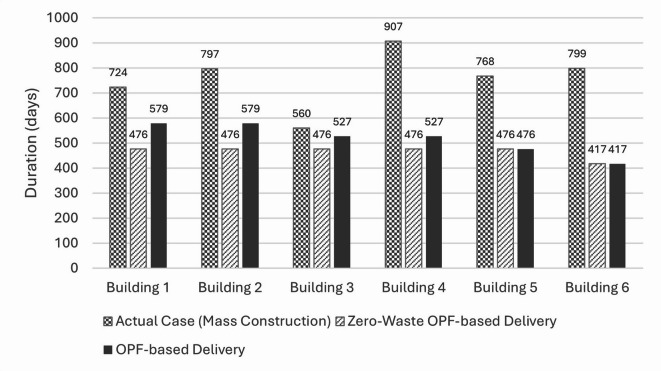
Duration of completing each building in all three construction delivery cases: (1) mass construction (actual case), (2) zero-waste OPF scenario, and (3) OPF scenario.

Comparing the three cases in terms of total project duration, it can be noticed that the OPF-based delivery scenarios required more time than the actual case of mass construction. This outcome is expected, as the batches start one after the other in the OPF-based delivery. However, when comparing the three cases in terms of the duration of each building individually, as shown in Fig. 4, it has been noticed that each building in the actual case consumed much more time due to delays and financial interruptions. For instance, while the 4th building required only 476 days to be completed under the zero-waste assumption, it actually took 907 days in reality (a delay of 431 days). On the other hand, it took 527 days to be completed using the OPF-based delivery under the assumption of zero delays due to financial problems and elimination of delays due to defected works by 50% in the 2nd batch and 100% in the 3rd batch.

**Fig. 5:**
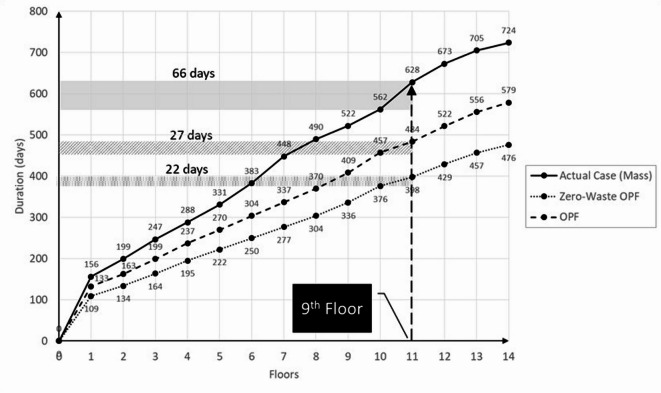
Repetitive schedule of Building 1 in all three construction delivery cases: (1) mass construction (actual case), (2) zero-waste OPF scenario, and (3) OPF scenario.

To further analyze the performance at the building level, Fig. 5 below shows the repetitive schedule of building 1, as a sample, in all three cases to visualize the progress across the floors. It can be noticed from the slope of the flow lines that the actual case consistently consumed more time to complete each floor compared to the OPF-based scenarios, due to the encountered waste in terms of rework and idleness. For instance, the 9th floor required 66 days in the actual case as opposed to 27 days in the OPF-based scenario and 22 days in the ideal zero-waste OPF-based scenario.

### Construction performance comparison: cost at completion

Similarly to the time-based comparison, the three cases will be compared based on the cost at completion. The cost was computed for each floor, by summing up the costs of the resources required to complete one floor (materials, crew wages, and machinery costs). However, as previously mentioned in the case study description, Egypt has suffered from economic instability during the project time period leading to huge price fluctuations. Therefore, it was essential to include the changes in the materials’ prices, crew wages, and machinery costs over time when estimating costs in the comparative analysis.

**Fig. 6:**
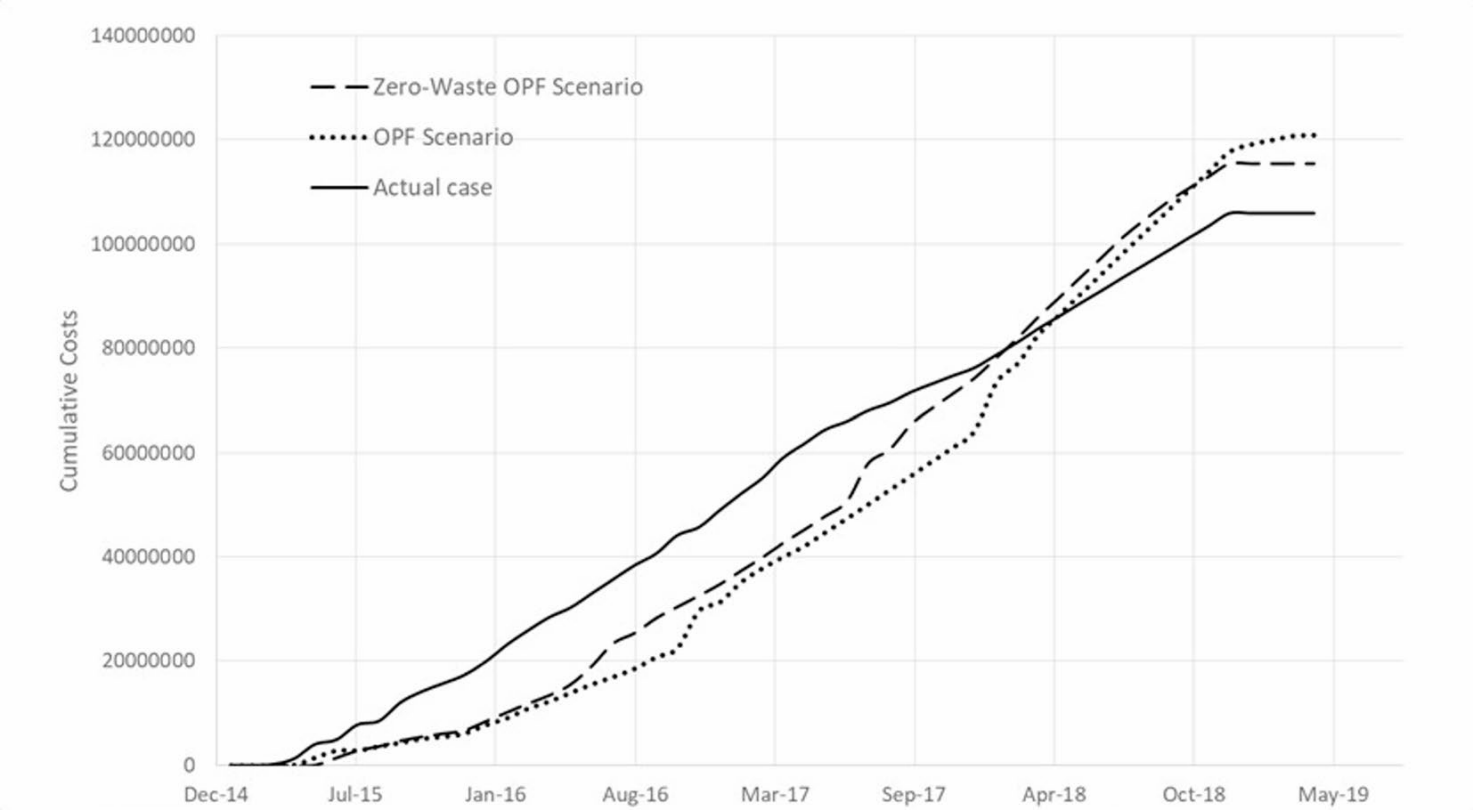
S-Curves for the project’s cumulative costs in all three construction delivery cases: (1) mass construction (actual case), (2) zero-waste OPF scenario, and (3) OPF scenario.

Figure 6 below shows the developed s-curves for the project’s cumulative costs in all three cases. It can be noted that the cumulative costs by the end of the project using mass construction is less than the OPF-based scenarios, giving an illusion of being a better method. However, this difference is due to the time difference between the execution dates of the activities in all three cases. The cost of executing an activity at a later date due to delivering the project in batches was affected by the increase in prices over time. Moreover, it can be noticed that mass construction led to excessive expenditures during the period from July 2015 up to September 2017, almost 2 years, in comparison with the OPF-based scenarios. This is due to the excessive cost waste as a result of rework.

### Economic feasibility analysis

Despite the fact that mass construction led to less total completion time and cost, it is not sufficient to confirm its outperformance over the proposed OPF-based delivery. One of the major pitfalls that developers can fall for is assuming that minimizing the costs would make the project economically feasible. In Lean philosophy, managers are encouraged to base their decisions on a well-articulated long-term vision, even if those decisions contradict what might be financially beneficial in the short term^38^. Therefore, an economic analysis was conducted to evaluate the revenues from selling the units against the incurred costs over time to determine which delivery method is more economically feasible and avoid financial losses.

Revenues are highly dependent on the adopted selling strategy. If mass construction is used, then the developer would sell large number of units to cover the expenses. If the units are constructed in batches, then the developer would sell the units in batches as well. Since the selling unit price changes over time, the revenues $\:{R}_{t}$ at any given time (*t*) are calculated as follows:

5 $$\:{R}_{t}={AoS}_{t}\times\:{UP}_{t}$$

6 $$\:{AoS}_{t}={\text{\%}S}_{t}\times\:A$$

Where, $\:{AoS}_{t}$ is the area (square meter) offered for sale at a given time *t*;$\:\:{UP}_{t}$ is the unit price per square meter at a given time (*t*); $\:A$ is the total area under construction which is function of the number of buildings, the number of floors per building, and the floor area; and $\:{\text{\%}S}_{t}$ is the percentage offered for sale at a given time (*t*) of the total area.

#### Mass selling strategy

In the actual case, the developer used mass selling strategy to accommodate mass construction. The developer sold a large percentage of the housing units before construction begins in order to secure proper funds to cover the construction expenses. For instance, the sales in the project started in January 2015, while the actual construction started in March 2015. Table 2 shows over time: (1) the sale price per square meter, (2) the percentages of the total area offered for sale, (3) the total area offered for sale, and (4) the sales revenues. It can be noted from the table that the unit price at the beginning of the project, according to the real estate housing market, was 3,500 EGP, and kept increasing until it reached to 13,000 EGP at the end of the project. The developer sold 60% of the project at low sale prices of 3,500 EGP and 5,000 EGP and only 5% of the project was sold at sale price of 13,000 EGP.

**Table 2:** Selling strategy in the case of mass construction.

Sale date	Jan 2015	Jul 2015	Apr 2016	Jan 2017	Jul 2017	Dec 2017	Feb 2018	Dec 2018
Price (EGP per m^2^)	3500	5000	6000	7000	8000	9000	11,000	13,000
Percentage of units offered for sale	40%	20%	10%	10%	5%	5%	5%	5%
Total area offered for sale (m^2^)	17,640	8820	4410	4410	2205	2205	2205	2205
Revenues (000s) EGP	72,324	51,660	30,996	36,162	20,664	23,247	28,413	33,579

#### OPF-based selling strategy

In the OPF-based scenarios, the buildings were constructed in three successive batches of 2 buildings at a time. Therefore, the selling strategy in both scenarios was assumed to follow the batch construction; the developer would start selling units in each batch right before the construction of each. It has been assumed that the percentages of housing units offered for sale in each batch follow the actual case percentages. Similar to Tables 2 and 3 shows over time the sales revenues generated using OPF-based selling strategy.

**Table 3:** Selling strategy in the case of OPF-based construction delivery.

Sale date	Jan-15	Jul-15	Apr-16	Jan-17	Jul-17	Dec-17	Feb-18	Dec-18	Feb-19	Jun-19
Price (EGP per m^2^)	3500	5000	6000	7000	8000	9000	11,000	13,000	15,000	15,000
Percentage of units offered for sale per batch 1	40%	`20%	10%	10%	10%	10%				
Percentage of units offered for sale per batch 2			40%	20%	10%	10%	10%	10%		
Percentage of units offered for sale per batch 3		`			40%	20%	10%	10%	10%	10%
Total area offered for sale (m^2^) per batch 1	7056	3528	1764	1764	1764	1764				
Total area offered for sale (m^2^) per batch 2			7056	3528	1764	1764	1764	1764		
Total area offered for sale (m^2^) per batch 3					6552	3276	1638	1638	1638	1638
Cumulative % sold of the total project BUA	14%	7%	17%	10%	20%	13%	7%	7%	3%	3%
Revenues (000s) EGP	24,696	17,640	52,920	37,044	80,640	61,236	37,422	44,226	24,570	24,570

It can be noted from the table that in the OPF-based scenarios, as opposed to the actual case, the developer didn’t sell a large percentage of the project at the beginning of the project since only one batch of 2 buildings was being constructed at a time. Also, the developer was able to sustain almost 15% of the project to sell at an increased unit price of 15,000 EGP, since the project duration was extended to accommodate batch construction.

#### Cash flow analysis

Having the monthly costs incurred and the sales revenues in each case, net present value (NPV) method was used to evaluate the project’s economic feasibility using each delivery method^39^. Since the interest rate is not constant and changing over time, the NPV has been computed by discounting each cash flow at a given month (t) using the multiplication of ($\:1+{i}_{t}$) from t = 1 up to t month [e.g., ($\:1+{i}_{1}$) × (1+10%) × (1+12%) × (1+$\:{i}_{t}$)], as shown below in Eq. (7).

7 $$\:NPV=\:\sum\:_{t=1}^{T}\frac{{R}_{t}}{\prod\:_{t=1}^{t}{(1+{i}_{t})}^{\:}}-\sum\:_{t=1}^{T}\frac{{C}_{t}}{\prod\:_{t=1}^{t}{(1+{i}_{t})}^{\:}}$$

Where, $\:{R}_{t}$ is the revenues at a given time *t*, $\:{C}_{t}$ is the total costs incurred at a given time *t*, $\:{i}_{t}$ is the interest rate at a given time *t*, and T is the time horizon over which the economic analysis has been conducted.

**Fig. 7:**
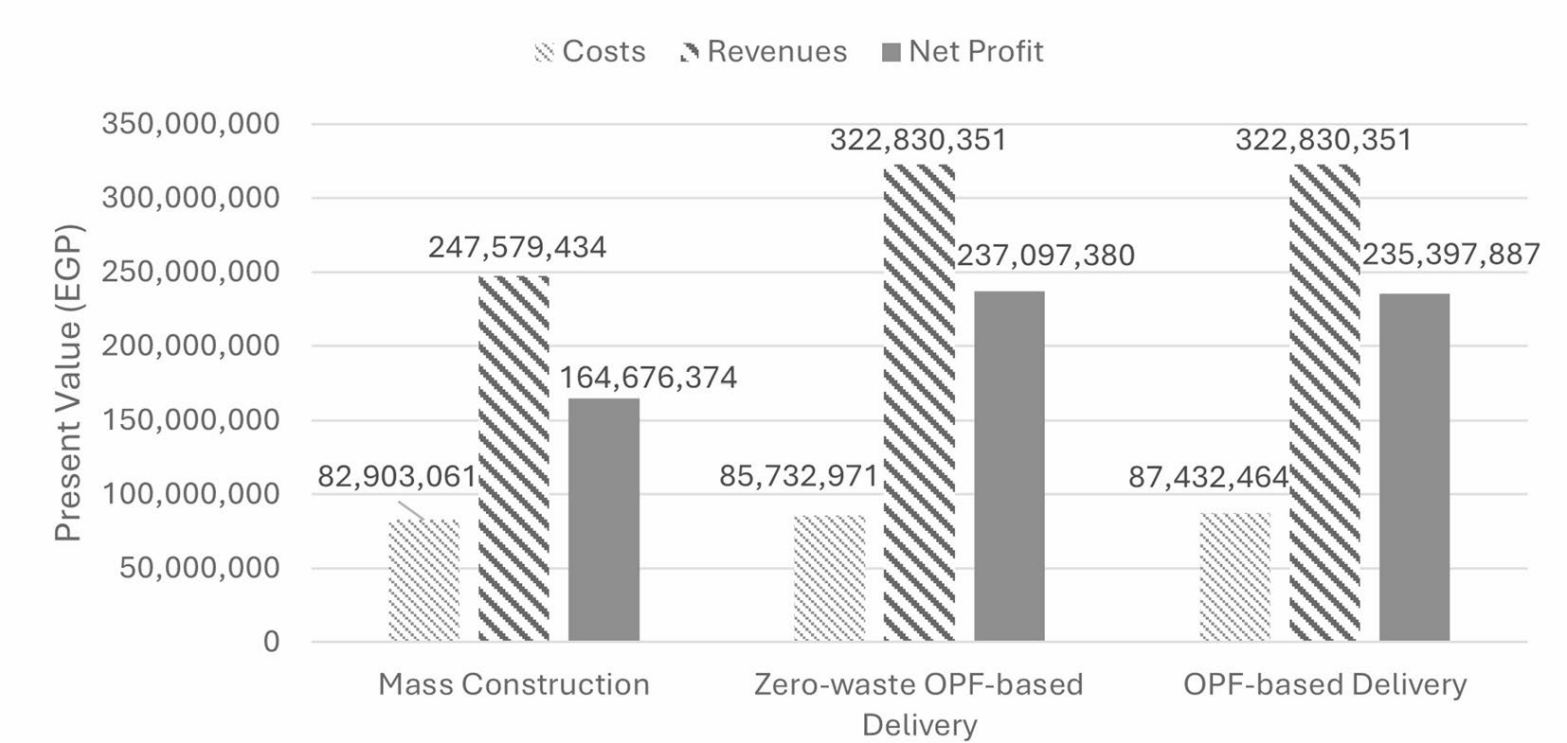
Comparison between mass construction and OPF-based construction delivery methods in terms of present value of costs and revenues and net profit.

Figure 7 shows the results of the comparative analysis in terms of the present value of the total costs and revenues, and the net present value (net profit). It can be noted that despite the fact that mass construction achieved the least costs, yet it achieved the least net profit. This is due to the adopted selling strategy of selling mass units upfront to secure funds for the large amounts of work running at the same time, and subsequently missing the opportunity of selling units at higher sales prices.

## Discussion

According to the conducted comparison with respect to the construction performance, it was found that the total cost in the ideal (zero-waste) and non-zero waste OPF-based cases was higher than the actual case by only 3.4% and 5.5%, respectively, due to the excessive cost waste resulting from rework in the actual case. While for the project duration, it was longer in the ideal (zero-waste) and non-zero waste OPF-based cases than the actual case by almost 27% and 50%, respectively. This is because the six buildings were delivered in three successive batches of two buildings rather than in one batch. The longer duration in the non-zero waste OPF-based case is due to incorporating 100% and 50% of the actual delays in the first and second batches, respectively, and 0% of the delays in the third batch. This assumption could be debatable and considered conservative or pessimistic from the perspective of lean philosophy, as it misses out on the full benefit of batch production, which is principally aimed at eliminating waste. However, it was necessary to examine the potential benefit of adopting batch construction even under these conservative assumptions. A sensitivity analysis could be conducted, including several combinations of the percentage of delay incorporated in each batch. However, that would be time-consuming and beyond the scope of this research paper which advocates for the potential benefit of using OPF-based construction in practice.

On the other hand, it was found that OPF-based construction helped reduce time waste and WIP in delivering each building. Time waste was reduced, on average, by 60% and 30% across the six buildings in both cases of zero-waste and non-zero waste OPF-based construction, respectively. According to the conducted comparison with respect to the economic worthiness, it was found that the OPF-based construction helped achieve higher profitability than the actual case by, on average, almost 44% and 43% in both zero- and non-zero waste OPF-based cases, respectively. This is due to the adopted OPF-based selling strategy that allows selling units in synchronization with the batch under construction. Accordingly, the conducted comparative analysis between delivering the project using mass construction and OPF-based construction confirms the potential benefit of adopting the lean concept of One-piece Flow for delivering MHPs.

This case-based research is limited to investigating the applicability of adopting batch production and One-piece Flow lean concepts only in mass-housing developments using traditional construction methods. Moreover, it used a single actual case study to investigate the impact of mass construction versus OPF-based construction. Therefore, future works may include further experimentation using additional studies and different types of projects, as well as further examination of the benefits of OPF-based delivery in incorporating the end-user feedback into batches that haven not yet started. Future works may also include further investigation of the impact of OPF-based construction on the crews’ productivity and skill development.

## Conclusion

This case-based research investigated, using a real case study, the applicability of adopting the lean concept “One-Piece Flow” (OPF) to deliver MHPs instead of mass construction, which is commonly used to expedite the completion of projects. One-Piece Flow allows production in small batches to maintain the flow of product from one process to another without any interruption, idleness, or large work in progress. Therefore, an OPF-based construction delivery is proposed in this research and evaluated against mass construction using a comparative analysis with respect to: (1) construction performance, and (2) economic worthiness. The case study used in this research is for a MHP located in Egypt that involved mass construction of six identical buildings concurrently. Two what-if scenarios were developed, in which OPF-based construction was used to deliver the project in three successive batches of two buildings. The first scenario assumed ideal working conditions on-site by taking full advantage of batch construction, while the second assumed more conservative conditions. The results of the analysis showed the potential of the OPF-based construction delivery method to achieve higher profitability than mass construction, despite requiring more time and cost to complete the project. Moreover, it minimizes time and cost waste in delivering each individual building. In essence, this case-based research contributes to the body of knowledge on lean construction. It proved the potential benefit and applicability of adopting the One-piece Flow lean concept in the construction industry for mass-housing developments.

## Data Availability

Data generated or analyzed during the study are available from the corresponding author by reasonable request.
